# Faster Results, Better Care? Impact of Meningitis/Encephalitis Syndromic Panel Testing on Pathogen Detection and Hospital Outcomes Beyond CSF Culture: A Literature Search for Diagnosticians

**DOI:** 10.3390/diagnostics16050691

**Published:** 2026-02-26

**Authors:** Kayanne Toutounji, Jean-Marc T. Jreissati, Rami Mahfouz

**Affiliations:** 1 Faculty of Medicine, American University of Beirut, Beirut 110236, Lebanon; kmt07@mail.aub.edu (K.T.); jtj02@mail.aub.edu (J.-M.T.J.); 2 Department of Pathology and Laboratory Medicine, American University of Beirut, Beirut 110236, Lebanon

**Keywords:** meningitis, encephalitis, multiplex PCR, culture, outcomes

## Abstract

**Background:** Syndromic testing panels, such as the BioFire FilmArray^®^ Meningitis/Encephalitis (M/E) panel, have become essential in altering the way that central nervous system diseases are diagnosed in the rapidly changing fields of molecular diagnostics, infectious diseases, and neurology. Long turnaround times, minimal pathogen output, and the requirement for live organisms are some of the common limitations of traditional cerebrospinal fluid culture techniques. A potential addition to traditional diagnostics is the FilmArray^®^ M/E panel, which uses multiplex polymerase chain reactions to identify many diseases quickly and simultaneously in a very short time, which affects multiple outcomes for the patient. **Aims:** Despite the M/E panel’s considerable speed and detection benefits, there are some issues related to cost, erroneous findings, and contextual interpretation. **Methods:** This narrative review highlights fundamental research and meta-analyses that have studied the FilmArray^®^ M/E panel’s practical performance while comparing its diagnostic accuracy, clinical impact, and cost-effectiveness with the CSF culture. The latter occurs across different demographics and contexts. **Results:** Different studies have demonstrated that the M/E panel significantly shortens hospital stays, decreases unnecessary antibiotic usage, and speeds up the diagnosis of meningitis or encephalitis. Nonetheless, the necessity for cautious diagnostic management and supplementary testing strategies is underlined as there exist variations in sensitivity and specificity across pathogens, especially in viral ones. By facilitating quick, focused, and data-driven treatment for patients, the BioFire FilmArray^®^ M/E panel provides an advancement in meningitis and encephalitis diagnostics, which is consistent with the concept of precision medicine. **Conclusions:** To adequately guarantee fair and efficient results, its best application into clinical practice requires integration with clinical judgment, conventional culture techniques, and economic optimization strategies.

## 1. Introduction

Meningitis and encephalitis (M/E) are potentially fatal diseases that require prompt diagnosis and treatment to avoid serious consequences, such as irreversible brain damage or even death. The foundation of pathogen detection has been established by conventional diagnostic techniques, such as singleplex polymerase chain reaction (PCR), Gram stain, and cerebrospinal fluid (CSF) culture. Yet, some of these techniques can take up to 5.6 days for results, which can cause a significant delay in therapeutic interventions, affecting prognosis [1].

In fact, the disadvantages of traditional methods are worsened by different elements, such as previous antibiotic use, which can potentially sterilize CSF samples and lower the microbial load below levels that could be detected. In this case, the latter would lead to false-negative results [1]. Furthermore, there are logistical delays associated with exporting PCR tests to reference laboratories, which could affect patient outcomes by increasing turnaround time (TAT). Moreover, traditional techniques are sometimes incapable of identifying fastidious or non-culturable infections and may have a low sensitivity in patients who have already received treatment [2].

The BioFire FilmArray^®^ M/E panel was chosen for this study because of its extensive clinical use, solid validation evidence, and accessibility to real-world performance data, all of which make it an excellent platform for targeted analysis and comparison. As a first-line molecular diagnostic tool, this panel has been used in numerous hospitals and laboratories throughout the world. It offers significant real-world data on performance, clinical implementation experience, and documented impacts on patient management, such as shorter antimicrobial therapy and diagnosis times [3,4].

As the first FDA-cleared CSF panel for M/E, the FilmArray M/E panel detects a broad range of bacterial, viral, and fungal CNS pathogens, establishing a benchmark for subsequent cassette-based multiplex diagnostics [3]. Multicenter evaluations comparing the QIAstat-Dx M/E panel with existing syndromic assays have noted the FilmArray M/E panel’s high sensitivity and specificity relative to CSF and blood cultures and other molecular reference methods [5]. Its rapid run time of approximately one hour enables prompt, targeted treatment decisions, influencing antimicrobial therapy choice and duration, and supporting more effective treatment strategies [3,4]. In one study, use of the panel was also associated with a reduction in average hospital length of stay (LOS) [3]. The FilmArray^®^ M/E panel represents a significant paradigm shift in the diagnosis of CNS infections, allowing close-to-patient, rapid assessment of a wide range of pathogens. Its simple testing process enables deployment in diverse care settings, including community and rural hospitals [4]. For young infants, simultaneous testing for bacterial and viral agents may facilitate more targeted use of antibacterial and antiviral therapies. Immunocompromised patients are also likely to benefit, as 5–10% of transplant recipients experience CNS infections manifesting as meningitis, encephalitis, or brain abscess [4]. However, each laboratory should evaluate the clinical scenarios and patient populations best suited for FilmArray^®^ M/E testing. As adoption increases, the panel’s clinical performance and associated impacts on patient care can be more systematically evaluated [3,5].

**Figure 1:**
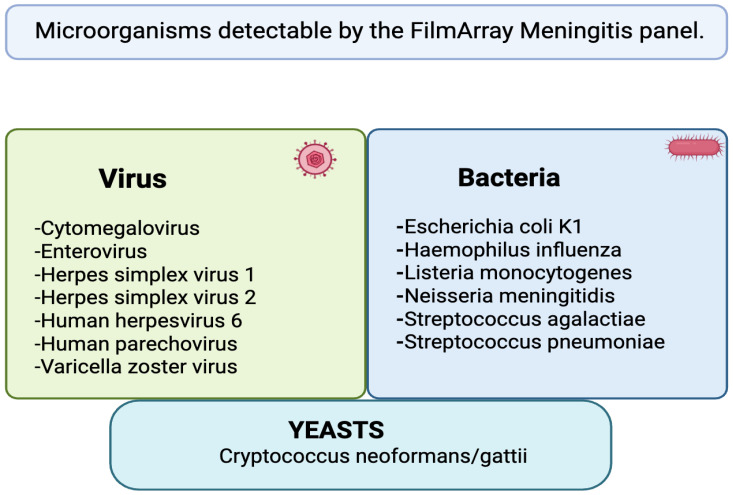
Microorganism detected via Biofire Film Array. Generated through the Biorender software (Biorender 2025) available via the following link: https://app.biorender.com/illustrations/6903bf756de958b87e770f09, Access date: 20 December 2025.

The BioFire FilmArray^®^ M/E panel (BioFire Diagnostics, Salt Lake City, UT, USA) is a quick, FDA-approved syndromic multiplex PCR test that can detect more than 90% of the most prevalent M/E pathogens and reports results in about an hour [6]. Six bacteria (*Escherichia coli K1*, *Haemophilus influenzae*, *Neisseria meningitidis*, *Streptococcus pneumoniae*, *Streptococcus agalactiae*, and *Listeria monocytogenes*), one yeast (*Cryptococcus neoformans/gattii*), and seven viruses (*enterovirus*, *HSV-1*, *HSV-2*, *VZV*, *CMV*, *HHV-6*, and *human parechovirus*) are among the 14 targets found in CSF [7]. Nowadays, it is usually used in conjunction with traditional microbiology and clinical evaluation to produce quick, useful findings that can direct focused therapy and cut down on unnecessary empirical treatment [7]. A representation of the latter is available in Figure 1.

According to some authors, the implementation of the FilmArray^®^ M/E panel has shown encouraging outcomes, such as an increased diagnostic precision and significant cost savings by reducing unnecessary hospital stays and empirical treatments [1]. For example, with this panel, studies highlight a 43 h decrease in antimicrobial duration and an average 39 h decrease in acyclovir use [8]. Furthermore, shorter hospital stays (with a mean reduction of 15.5 days in some cohorts) and lower morbidity and mortality in delayed treatments are correlated with faster diagnosis [1,8]. In addition, when the panel’s results are negative, physicians may de-escalate or stop using empirical treatments, such as acyclovir or broad-spectrum antibiotics. Thus, there is potential for improved patient care and resource usage by comparing the effects of the FilmArray^®^ M/E panel vs. conventional CSF culture methods on pathogen identification efficiency and hospital outcomes [1].

To maximize cost-effectiveness and diagnostic accuracy, their adoption necessitates a careful integration with current workflows [9]. Nevertheless, not all findings are consistent, the topic is rapidly evolving, and there may be differences in hospital settings (ICU vs. general ward, or pediatric vs. adult patients). There may also be differences in terms of laboratory protocols and populations. Moreover, variables such as TAT and length of stay (LOS) could be affected by different factors, such as the timing of presentation and clinical decisions that are made. It is also possible that some research studies based on commercial panels could be influenced or funded by manufacturers, which brings biased results. Therefore, the goal of this narrative review is to evaluate the impact of M/E panels on diagnostic accuracy as well as clinical outcomes. These also include LOS and antimicrobial use compared to conventional methods, such as CSF culture.

Thus, this narrative review aims to answer the following clinical questions: Among patients with suspected M/E, does FilmArray^®^ M/E panel, as opposed to traditional diagnostic methods, such as CSF culture, enhance the detection of pathogens and shorten the time for diagnostic test results? Additionally, what are its implications on downstream hospital outcomes, such as LOS, antimicrobial and antiviral use, as well as clinical decision-making in a variety of patient groups and clinical care environments?

## 2. Methods

A comprehensive search of the literature was carried out in PubMed and MEDLINE using terms such as “FilmArray M/E panel,” “diagnosis of meningitis,” and “clinical outcomes” to find relevant studies. To concentrate on solid clinical and diagnostic investigations, only English-language publications were considered, and case reports, case series, and conference abstracts were not considered. To guarantee that current diagnostic methods and clinical procedures were included, papers that were more than 15 years old were also eliminated. The search results and study selection were independently cross-checked by two authors. Any discrepancies regarding inclusion were resolved through discussion as well as consensus. Therefore, 99 articles were identified for screening, and 51 were included in this manuscript. The inclusion criteria consisted of research that reported pathogen detection rates (e.g., sensitivity/specificity/positive predictive value [PPV]/negative predictive value [NPV]), clinical outcomes (e.g., length of stay [LOS], duration of antimicrobial therapy), and demographic information (e.g., age, gender, cerebrospinal fluid [CSF]/blood culture/urine culture results). Only studies involving human subjects were considered. The primary outcomes were the accuracy of pathogen detection and LOS, while secondary outcomes included time to diagnosis (or TAT) and mortality rates.

## 3. Results

### 3.1. The Diagnostic Performance of Meningitis Panels

**Figure 2:**
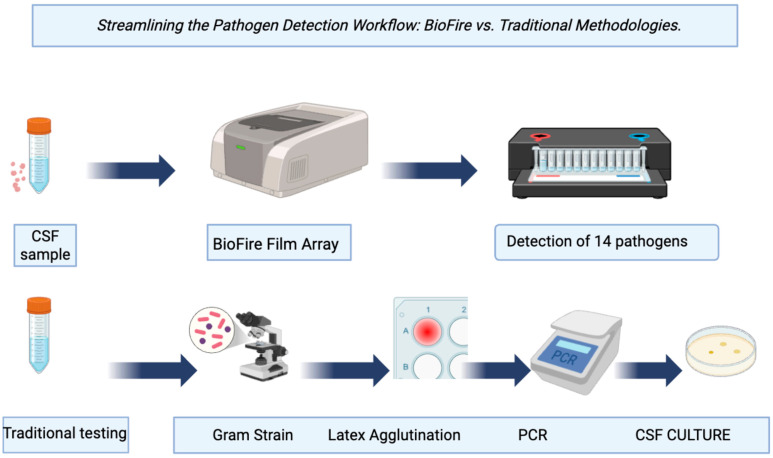
Biofire FilmArray^®^ detection workflow compared to traditional methods. Generated through the Biorender software (Biorender 2025) available via the following link: https://app.biorender.com/illustrations/6903bf756de958b87e770f09, Access date: 20 December 2025.

Numerous studies have assessed the diagnostic performance of M/E panels (such as sensitivity, specificity, negative predictive value (NPV) and positive predictive value (PPV)), and the results have been mostly consistent with traditional techniques. For instance, when it comes to identifying pathogens, such as *Escherichia coli*, *Haemophilus influenzae*, *Neisseria meningitidis*, and Varicella zoster virus (VZV) in CSF samples, the FilmArray^®^ M/E panel demonstrated 95% agreement with conventional PCR approaches [1]. Nonetheless, depending on the pathogen, there are some differences in sensitivity and specificity. In contrast to reference laboratory PCR, the FilmArray^®^ M/E panel demonstrated lower sensitivity (86%) for some viral pathogens, including Human herpes virus 6 (HHV-6), even though bacterial targets typically exhibit high concordance [1]. An image depicting the panel’s detection workflow compared to the traditional methods is available in Figure 2.

### 3.2. The Pathogen-Specific Performance and False Results

More specifically, the M/E panel’s discovery of HHV-6 in neonates frequently indicates chromosomally integrated virus (ciHHV-6) or silent congenital infection rather than acute illness. In fact, HHV-6 was found in 2.1% (5/242) of 242 newborns in the cohort, and none of them needed antiviral treatment. A benign illness known as ciHHV-6 is suggested by high viral loads (>999,000 copies/mL) in plasma or CSF [10]. In other words, since the viral DNA is a component of the host DNA, PCR testing can identify extremely high viral loads, particularly in whole blood, even when the infection is not active. This may result in incorrect diagnoses of acute HHV-6 infection, particularly in patients undergoing testing for encephalitis or meningitis. However, there was little correlation between HHV-6 identification and symptoms (e.g., 1/7 newborns experienced rash), highlighting the necessity for cautious interpretation to prevent unnecessary interventions. In this case, to rule out ciHHV-6, it is important to perform HHV-6 PCR quantification (such as plasma testing) for newborns with high CSF viral levels [10].

The FilmArray^®^ M/E panel may also have a large rate of false positives (such as Herpes simplex virus 1 (HSV-1) misidentification), and its potential low false-positive rate may result in the use of incorrect antiviral medication [2,3]. In their multicenter trial, for example, some authors reported 12 unresolved false-positive HSV-1/2 results, highlighting the necessity of interpreting FilmArray^®^ M/E panel results with caution [3]. Except for *Streptococcus agalactiae* (because of a false-negative result) and HHV-6, the M/E Panel demonstrated excellent concordance for most pathogens. The necessity for clinical correlation is highlighted by the fact that *S. pneumoniae* and viral targets were the main sources of false positives [3].

Similar results were found by some authors, who found that this panel has an overall sensitivity of 90% and specificity of 97%. Prior to adjudication, there was a high false-positive rate (17.5%) for *Streptococcus pneumoniae*, frequently as a result of contamination or inappropriate handling [6]. Specificity increased during adjudication, and lingering false positives indicate that interpretation should be carried out with caution [6]. As for *Group B Streptococcus or Streptococcus Agalactiae*, after adjudication, there was a 15.4% false-positive rate, suggesting possible contamination or cross-reactivity [6].

False negatives were most common with *Cryptococcus neoformans* and HSV-1/2, highlighting the necessity of additional testing in high-risk situations. Since cryptococcal antigen (CrAg) remains after treatment, false negative results frequently occur in patients receiving antifungal therapy [6,11]. False negatives are also present in patients with low fungal burdens [11]. Furthermore, discordant analysis improved overall reliability by lowering false negatives by 31.8% and false positives by 65.2% [6]. Overall, CrAg testing is still essential for diagnosis in high-risk Human immunodeficiency virus-positive patients (HIV-positive), despite the M/E Panel’s 99.9% NPV for culture [11,12,13].

According to different studies, the BioFire FilmArray^®^ M/E panel has significant limitations in detecting pathogens, which affects its reliability as a standalone diagnostic method, particularly for viral and opportunistic organisms [1,13,14]. One of the authors, for instance, found that the sensitivity for viral targets (such as enterovirus and HHV-6) was only 86%, with false negatives resulting from inadequate detection thresholds or low viral loads in the CSF. More concerningly, the panel shows ongoing challenges in detecting high-risk bacterial and fungal pathogens [1]. A comparison across different studies of the specificity and sensitivity of pathogen detection through the M/E Panel is available below in Table 1 [2,3,4,6,7,11].

In another study, a 69-year-old man presenting with classic rhombencephalitis (evidenced by MRI brainstem enhancement, nystagmus, and ataxia) received two successive negative M/E panel results for *Listeria monocytogenes*, despite having had no previous antibiotic treatment. This led to a delay in treatment until the administration of steroids resulted in clinical deterioration and subsequent confirmation through PCR or culture [14].

Similarly, another author reported a false-negative M/E panel result for *Cryptococcus neoformans* in an immunocompromised patient (with mantle cell lymphoma) suffering from subacute meningitis, even with pronounced CSF pleocytosis (560 WBC/mm^3^) and elevated protein levels (261 mg/dL) [13]. *Cryptococcus neoformans* was ultimately identified only after serum/CSF cryptococcal antigen testing (with a titer exceeding 1:2560) and culture, highlighting the panel’s insufficient sensitivity for fungal detection, particularly in HIV-negative patients, whose antigen levels may differ [13]. On the other hand, when compared to traditional techniques like CSF culture, the QIAstat-Dx M/E Panel exhibits excellent diagnostic performance, with a sensitivity of 96.4% and specificity of 95.2% [2]. It can identify bacteria (such as *Streptococcus pneumoniae*, *Listeria monocytogenes*), viruses (like HSV-1/2, VZV), and fungi (like *Cryptococcus neoformans*) [2]. Furthermore, the high PPV and NPV of the QIAstat-Dx ME Panel (96.4% and 95.2%, respectively) reduce the need for unnecessary procedures and hospital stays, which may enhance patient outcomes and lower medical expenses [2].

A comparison across different studies of the types of error per pathogen is available in Table 2 [3,6,10,11,13,14].

### 3.3. The Value of Ancillary Testing

When CSF culture results are negative or unclear, blood and urine cultures are useful in the diagnosis of bacteremia. For example, some scientists discovered a case of *Escherichia coli* meningitis that was overlooked by the FilmArray^®^ M/E panel but discovered through CSF culture [16]. Positive urine cultures and renal imaging further supported their findings. This emphasizes how crucial it is to use auxiliary tests to improve diagnostic precision, particularly in situations where CSF results are unclear or where previous antibiotic usage may have inhibited the identification of pathogens in CSF [16]. According to them, these multimodal testing techniques enhance the detection of systemic illnesses and direct focused treatment. For instance, blood and/or urine cultures can detect pathogens (e.g., *Escherichia coli* bacteremia with urinary tract involvement) or even confirm CSF findings when the latter tests are negative. Moreover, ancillary tests are essential for infection detection because prior antibiotic administration may lower CSF culture sensitivity. Furthermore, by optimizing microbiological diagnosis and informing treatment duration, the combination of CSF panels, blood, and urine cultures minimizes the unnecessary use of antibiotics [16]. A direct comparison between the M/E Panel and CSF culture methods is available in Table 3 [4,9,15,17,18,19,20,21,22,23].

### 3.4. The Impact on Clinical Outcomes and Timing

M/E panels greatly cut down on TAT and facilitate prompt clinical decision-making [1]. The same applies to the QIAstat-Dx M/E Panel discussed above, as both can target multiple bacterial, viral, and fungal pathogens in one single test [2,9]. Similarly, the latter panel provides results within 76 min [9]. These findings demonstrate significant advantages in antimicrobial stewardship. Some authors further shed light on the clinical value of stewardship in translating rapid diagnostics by assessing the M/E panel solely inside a structured diagnostic and antimicrobial stewardship program [25]. Their intervention included direct results transmission to healthcare teams, real-time stewardship evaluation, and Electronic Medical Record (EMR)-guided test ordering [25]. Enterovirus-positive patients had much shorter antibiotic durations under this approach, and the time to optimal antimicrobial therapy fell significantly [25]. Rather than relying solely on assay performance, these advances were specifically linked to stewardship-guided interpretation and action [25]. The median duration to optimal antimicrobial therapy dropped from 28 h before implementation to 18 h after implementation (*p* < 0.0001) [25]. The post-implementation group had a 13% increased chance of receiving the best antimicrobial treatment (adjusted hazard ratio 1.13; 95% CI 1.04–1.23) [25]. Patients with enterovirus had much shorter antibiotic duration [25].

It is important to mention that the clinical impact of rapid diagnostics depends highly on context and varies according to patient age, immune status, pathogen type, disease severity, as well as the presence or absence of active diagnostic and antimicrobial stewardship. As such, reduction in hospital LOS and antimicrobial exposure are not uniformly observed across studies and have to be analyzed within the clinical and institutional context in which the M/E panel is used [8].

### 3.5. The Effect of Antimicrobial Use

According to a comprehensive analysis, 75% of the included trials demonstrated that the FilmArray^®^ M/E panel reduced acyclovir use by an average of 39 h [8]. This decrease is explained by the panel’s quick TAT, as mentioned above and excellent sensitivity for identifying viral pathogens, which enables physicians to stop empirical acyclovir treatment earlier after ruling out HSV. Thus, faster results lead to earlier targeted therapy [26].

Other studies similarly demonstrate that, in comparison to traditional testing methods, the M/E panel’s quick TAT enables clinicians to stop acyclovir earlier when findings are negative [27,28]. In a multicenter retrospective study, authors discovered that once the M/E panel was implemented, the median intravenous (IV) acyclovir duration dropped from 41.6 h to 30.8 h (*p* < 0.01) [27]. Faster test TAT (from 37.9 h to 6.2 h) were credited with this decrease, which allowed for the stop of unnecessary therapy [27]. In a similar vein, other authors found that quick viral pathogen detection significantly reduced the duration of acyclovir in pediatric patients, especially young pediatric patients, from 3 days to 1 day (*p* < 0.001) [28]. However, clinical impact can differ according to patient groups, institutional protocols, and the presence or absence of diagnostic stewardship (for example, real-time physician notification).

Likewise, 44% of studies saw a reduction in the length of time spent on broad-spectrum antibiotics, with an average reduction of 43 h [8]. Nonetheless, the effect on antibiotic use was less consistent because, especially in pediatric and immunocompromised patients, physicians frequently prolonged medications pending culture findings or out of worry for non-CNS infections [8]. Nevertheless, results are mixed. In fact, in a large pre-post study, researchers discovered that the M/E panel decreased the median empirical antibiotic duration from 34.7 h to 12.3 h [29]. Especially in pathogen-negative cases, the quick TAT (2.6 h vs. 71.3 h for standard viral PCR) allowed for the earlier cessation or de-escalation of antibiotics [29]. On the other hand, some authors found no discernible variation in the length of hospital stay or antibiotic duration (median three days before vs. after the M/E panel, *p* = 0.571) [30]. Despite quick diagnoses, 78% of patients with negative panel results were still given empiric antibiotics, indicating that clinicians may be reluctant to stop treatment [30]. Regardless of M/E panel results, ongoing empirical antibiotic therapy and observation may be prudent in febrile neonates and young children without localizing indications of illness. This reflects cautious and guideline-concordant therapeutic practice rather than diagnostic failure [8].

Other researchers demonstrate, however, that the overuse of antibiotics might result in side effects, such as transaminitis and thrombocytosis [31]. In addition, the quick identification of viral pathogens by the meningitis panel not only reduces the usage of antibiotics and related hazards but also hospital-acquired infections and antibiotic resistance [31]. Yet, some factors have contributed to this variability in some studies [29,30]. For example, some concentrated on adults in the emergency department (ED), where a timely rule out of bacterial meningitis could speed up clinical decisions [29]. On the other hand, others observed larger populations where comorbidities or unclear diagnoses could make treatment take longer [30]. Furthermore, reductions without official stewardship treatments were reported, suggesting that clinicians were persuaded only by the panel’s results [29]. Yet, according to another researcher, without further stewardship initiatives, passive reporting of the panel’s results is not enough to alter prescribing practices [30].

### 3.6. The Effect on the Length of Stay at the Hospital

The patient population, stewardship policies, and baseline diagnostic capacity all affected the clinical impact of MEP implementation. Reductions in LOS were only seen when standard-of-care (SOC) testing for enterovirus and human parechovirus was carried out externally in Australian pediatric trials, where viral meningitis predominated. Centers with in-house molecular testing showed no LOS advantage. In a similar vein, a Colombian study carried out in a high-HIV-prevalence setting revealed no decrease in LOS or acyclovir duration after M/E adoption; HSV was only found in the M/E group, indicating frequent empirical antiviral treatment without microbiological confirmation under SOC settings. These results emphasize that while implementing M/E testing, institutions must take local epidemiology and current diagnostic infrastructure into account [8]. Across studies, approximately 40% of trials showed LOS reductions, although the benefit varied and differed among patient populations [8]. For instance, the review’s sole randomized trial found that replacing external PCR testing with M/E panel testing reduced the LOS for pediatric patients with viral meningitis by 3 days [8].

However, while recognizing its limitations in diagnosing specific pathogens, some scientists illustrated that the FilmArray^®^ M/E panel provides noteworthy clinical and economic advantages due to its significantly quicker TAT, especially in pediatric cases [1]. Their prospective research indicated that the panel delivered results in approximately 70 min, in contrast to 5.6 days required for traditional reference laboratory testing. This rapid result turnaround was directly linked to a 15.5-day average decrease in LOS for pediatric patients suspected of having meningitis/encephalitis. The swift TAT resulted in the earlier cessation of empiric antimicrobials in viral instances and more targeted treatment for bacterial infections, minimizing unnecessary treatments and complications [1].

### 3.7. The Pathogen Type and Clinical Impact

The type of pathogen found greatly affects the M/E panel’s utility and LOS. For instance, the M/E panel has a significant effect on lowering antiviral use and LOS for enterovirus meningitis. In fact, that same-day enterovirus PCR results considerably reduced LOS by 0.5 days and the duration of intravenous antibiotics by 0.67 days [32]. Although the M/E panel also allows for the de-escalation of empirical medicines for bacterial or fungal meningitis, its effect on LOS is less reliable when it comes to *Streptococcus pneumoniae* or *Cryptococcus neoformans* [33]. In their study, the authors discovered that the M/E panel raised the detection frequencies of bacterial pathogens (from 0.4% to 18.7%), but had no discernible effect on antibacterial days of therapy (DOT) or LOS [33]. This would explain how physicians are being cautious when stopping antibiotics in spite of negative M/E panel results, particularly in high-risk situations, as was seen above with other authors [30].

For low-sensitivity targets like viruses (86% sensitivity), *Listeria monocytogenes*, or *Cryptococcus neoformans*, where false negatives could lead to delayed treatment and extended LOS, the efficiency benefits of the panel may be compromised. Therefore, although the FilmArray^®^ M/E panel presents speed benefits with measurable outcomes, its implementation must consider the clinical context, and negative findings in high-risk patients (such as in immunocompromised individuals or those who have rhombencephalitis) should still prompt additional testing to avert critical diagnostic oversights [1].

According to the studies reviewed, the FilmArray^®^ M/E panel’s effect on LOS and ICU admissions varies considerably based on the type of infection and clinical situation. For viral CNS infections, the M/E panel greatly reduces LOS by facilitating swift pathogen detection and permitting early discontinuation of unnecessary antiviral treatments. Some authors demonstrated a 3-day decrease in LOS for pediatric cases of aseptic meningitis (from 8 days to 5 days), which was due to the rapid identification of enteroviruses and human parechoviruses, allowing for earlier cessation of empiric acyclovir and antibiotics [34].

Similarly, other authors indicated a significant reduction in acyclovir usage (from 6 days to 0 day), yet noted that overall LOS remained unchanged since antibiotic administration continued while awaiting culture results for potential bacterial infections [35]. On the other hand, bacterial or fungal infections showed limited reductions in LOS. Different authors observed no difference in LOS for adults with cryptococcal meningitis or bacterial infections (18 vs. 17 days), as the duration of treatment was reliant on clinical response and the length of antifungal or antibiotic courses, rather than the speed of diagnosis [12]. The risk of false negatives associated with *Cryptococcus neoformans* also required prolonged therapy until conclusive tests were completed [12].

Moreover, some observed no reduction in ICU stays, more particularly PICU stays (6 vs. 4 days, *p* = 0.36) [35]. In fact, ICU admissions were influenced by severity (e.g., status epilepticus or encephalopathy necessitating targeted temperature management), rather than delays in diagnosis [35]. Likewise, authors reported unchanged ICU stays (4 vs. 6 days, *p* = 0.502), highlighting that critical care requirements in immunosuppressed patients (e.g., HIV-associated cryptococcal meningitis) remained steady despite rapid diagnostic capabilities [12].

According to a study, the BioFire FilmArray^®^ M/E panel effectively detected *Cryptococcus neoformans* in CSF, with 88.9% (24 out of 27) of positive results validated as true positives through CSF culture, resulting in a PPV of 96.3% [36]. This rapid molecular test successfully identified cases of cryptococcal meningitis that may otherwise be overlooked, especially given the high rate of immunosuppression (51.9%) in this population. Although the research took place at a tertiary care center in Kentucky, its results emphasize the panel’s reliability for diagnosing cryptococcal infections. The high rate of CSF culture confirmations (88.9%) was linked to the usual absence of pre-treatment antifungals, unlike bacterial cases, where previous antibiotic use often diminishes culture yields. No false negatives were found for *Cryptococcus neoformans*, although a false positive (3.7%) was recorded in an infant who exhibited no clinical signs of meningitis [36]. These findings demonstrate the panel’s effectiveness in swiftly confirming cryptococcal meningitis, which is a significant benefit in environments where diagnostic cultures may experience delays or where prior treatment affects traditional diagnostic methods. Tuberculosis was not evaluated in this study [36].

#### 3.7.1. Age-Related Variations in M/E Panel Results

The M/E panel shows differing effects across age groups. For example, some authors found that infants exhibit the greatest benefits with the biggest decreases in LOS and duration of antimicrobial therapy [28]. Indeed, according to data, 51.5% of the patients in their cohort were under a year old, confirming that meningitis and encephalitis disproportionately affect younger children. This is in line with earlier reports that indicate infants are most vulnerable to CNS infections, especially bacterial meningitis [37].

This is probably because the panel can quickly identify viral diseases that are prevalent in this age range, which frequently result in unnecessary empirical treatment, such as enteroviruses and HHV-6 [28]. Increased virus loads and underdeveloped immune systems are probably the causes of greater positive rates in infants. Moreover, enteroviruses (EV) and human parechovirus are the predominantly detected pathogens in this population [38]. In fact, EV was the most frequently found pathogen (45.1%), and particularly in infants less than two months, parechovirus was the second most prevalent (19.6%) [38].

Additionally, the relationship between infections and age was examined. The two most common pathogens causing neonatal bacterial meningitis in a study were *S. agalactiae* and *E. coli K1*. Some observed the same outcome, namely that the M/E panel improved the identification of *E. coli* and *group B Streptococcus* in young children with meningitis. *S. agalactiae* and *E. coli* are the main etiological agents for neonatal bacterial meningitis infection, according to another research conducted in Australia, London, and Canada. In the 1–6 age group, *S. pneumoniae* was the most common pathogen isolated. This result was in line with a Korean study that found *S. pneumoniae* to be the most common etiologic pathogen outside of the newborn period [37].

The M/E panel demonstrates lower detection rates in older individuals and children, which probably reflects the wider differential diagnosis in these age groups (such as autoimmune encephalitis) [38]. *Streptococcus pneumoniae* emerged as the most commonly detected bacterial pathogen (13.7% of positive cases), whereas bacterial pathogens were more prevalent in older pediatric populations [38]. The need to take patient age into account when interpreting the results and making treatment decisions is shown by this age-related variation in pathogen distribution. However, there is limited data on gender with no significant trends reported.

#### 3.7.2. Economic Impact of the M/E Panel

Despite the extra expense of the M/E panel itself, research analyzing healthcare expenditure revealed either no discernible difference or lower implementation costs. Reduced usage of antibiotics and shorter hospital stays were the main drivers of savings [8]. For example, one study found that hospitalization costs decreased by CAD 2319 per case [8]. These results provide credence to the cost-effectiveness of the panel in particular clinical settings, especially when paired with diagnostic stewardship to reduce unnecessary testing. Similarly, in another study, the reduction in LOS through the M/E panel alone resulted in potential savings of EUR 114,000 in pediatric care and could amount to over EUR 2 million across the hospital by factoring in decreased diagnostic delays and resource use [1].

Nonetheless, according to two different studies, imposing restrictions on CSF tests poses significant obstacles to cost management [1,39]. Some authors found that confining CSF diagnostics to a specific panel, which includes a costly multiplex PCR test priced at USD 854, did not lead to decreased overall testing expenses or reduced antibiotic usage. Moreover, it may offset savings [39]. This lack of savings was mainly due to a marked increase in the usage of the expensive panel itself (rising from 61.8% to 84.7%), which counterbalanced the reduction in non-panel test orders [39]. In contrast, other authors reported notable savings (around EUR 114,000 in pediatric cases) linked to a decrease in LOS after optimizing tests, suggesting that financial advantages may hinge more on enhancing clinical results like LOS rather than merely limiting the range of tests [1].

These authors reported considerable cost reductions linked to the FilmArray^®^ M/E panel, mainly due to its faster turnaround time, which facilitated earlier clinical decisions and shorter hospital stays. Their study indicated a statistically significant average decrease in pediatric length of stay (LOS) by 15.5 days, leading to net savings of EUR 114,000 for the pediatric group alone [1]. When these findings were applied across the hospital, the overall reductions in LOS, unnecessary antimicrobial use, and additional testing resulted in potential savings of over EUR 2 million annually. Importantly, although the FilmArray^®^ M/E panel had higher direct testing costs (EUR 12,480 vs. EUR 2800 for 94 samples), these costs were largely compensated by downstream efficiencies: quicker cessation of unnecessary antivirals/antibiotics in confirmed viral cases (approximately 68% of positives), decreased ICU transfers, and elimination of extended diagnostic ambiguity [1].

## 4. Discussion

### 4.1. Limitations and Challenges

#### 4.1.1. Regional and Clinical Challenges

The Biofire FilmArray^®^ M/E Panel has a wide range of applications and effects depending on the location and available resources. The majority of research assessing it comes from wealthy nations, where quick TAT and sophisticated diagnostic facilities are more readily available [8].

Traditional diagnostics (such as CSF culture and Gram stain) frequently have limited sensitivity (only 2% positivity in one study) in low-income nations like Ethiopia because of poor infrastructure, a lack of reagents, and past antibiotic use [40]. For the first time in ordinary practice, the M/E panel discovered viral etiologies (57% of positives) and greatly enhanced pathogen detection (10% positivity), despite its high cost [40]. Moreover, low-resource countries tend to have high burdens of M/E [8].

High reagent costs (approximately USD 100 per test) and logistical obstacles to procurement are significant limitations that restrict sustainability. Nonetheless, even in labs with insufficient molecular expertise, the panel’s ease of use (no training required) and quick TAT (one hour) make it possible [40]. In addition, the M/E panel is useful for filling important gaps in pathogen detection in low-resource environments, when traditional techniques like CSF culture or external PCR referrals are unavailable [8]. Contrary to limited resources environments, tertiary centers have the possibility to use additional tests, including CrAg, to reduce the panel’s false-negative cryptococcal results [12].

#### 4.1.2. The Role of Antimicrobial Stewardship

Moreover, the M/E panel’s pathogen targets are in accordance with the epidemiology of North America and Europe, limiting its usefulness in endemic infections (such as arboviruses and Mycobacterium tuberculosis), which might not be included on the panel [8]. Future research in low- and middle-income countries (LMICs) is required to determine the panel’s cost-effectiveness and usage in these contexts, where it may offer an influence on outcomes of hospitals and antimicrobial stewardship. Hence, the impact may vary in terms of clinical and economic impact of the panel in areas with differences in epidemiological and economic profiles, further enhancing the need for context-specific implementation techniques.

Furthermore, the BioFire FilmArray^®^ M/E Panel has some serious drawbacks, especially with regard to false positives and false negatives. Nevertheless, it may not consistently detect infections of the central nervous system promptly. For example, in one case, despite having a high level of clinical suspicion, *Listeria monocytogenes* was not identified by the panel until the patient’s condition deteriorated [14]. Indeed, it is reported in the literature that the accuracy of the panel is decreased when minimal pathogen levels or regional infections are not included in the design of the panel, making it difficult to identify pathogens like Epstein-Barr virus (EBV), *Mycobacterium tuberculosis* and specific bacteria like *Escherichia coli* (K1 capsular antigen) and *Cryptococcus neoformans* [41]. Additionally, false positives may also occur. In fact, some reports highlighted that HHV-6 detection was more suggestive of a latent infection than an active illness, especially in immunocompromised patients [42]. One should further emphasize that even though the BioFire FilmArray^®^ M/E Panel is a helpful tool, it is preferable not to use it as the only diagnostic technique, depending on the clinical picture of the patient. One should thus use it with other tests (like imaging, serology, and cultures) while keeping in mind the local epidemiological patterns.

The BioFire FilmArray^®^ M/E panel’s ability to reduce the needless use of antibiotics is largely dependent on how well it is incorporated into clinical procedures and stewardship initiatives. Lack of structured education or stewardship interventions prevents the panel from reducing the number of days of therapy or the empirical antibiotic duration in adults with suspected meningitis [39]. While it has been shown that active stewardship initiatives, like real-time result notifications, physician education, and diagnostic algorithms, have been shown to reduce usage of antibiotics [39]. In some cases, the antimicrobial therapy was further prolonged by positive Gram stains or cultures, even for non-panel pathogens like Aspergillus, which highlights the panel’s disadvantages in terms of thorough pathogen detection [39].

### 4.2. Future Directions

Future work should prioritize (1) diagnostic stewardship models, (2) LMIC implementation, and (3) algorithm-guided testing using CSF biomarkers.

#### 4.2.1. Diagnostic Stewardship and Optimization Strategies

Because M/E testing has had a better yield in general, it is crucial to give it priority in children with suspected CNS infections [38]. Furthermore, additional testing for non-panel pathogens (such as *Mycobacterium tuberculosis* in endemic areas) would be necessary due to reduced detection in older age groups in general [38].

The BioFire FilmArray^®^ M/E panel is also one of the multiplex PCR panels that can be integrated into diagnostic stewardship programs. Research highlights the importance of integrating these panels with pleocytosis criteria for CSF to enhance testing procedures and reduce pointless steps [8]. It has been shown that diagnostic stewardship can increase test yield while reducing inappropriate use. Examples of this include restricting testing to high-risk patients, such as those with immunocompromised conditions or CSF pleocytosis. For instance, some authors highlighted that age-specific CSF pleocytosis thresholds were found to reduce test utilization by 43% and increase the diagnostic yield of M/E panel by 62% [43]. But, it’s important to exercise caution when implementing these limitations to prevent missing serious infections, particularly in immunocompromised patients whose CSF results might not exhibit normal trends [7]. To guarantee the appropriate use of these diagnostic panels in a variety of clinical settings, future research should work to develop standardized stewardship models. Furthermore, one should combine the implementation of M/E panel with audit-and-feedback initiatives and diagnostic algorithms [39].

#### 4.2.2. The Implementation in Low-Resource Settings

In LMICs, the introduction of syndromic testing panels, such as the QIAstat-Dx M/E panel, calls for cost-effective approaches that balance accessibility with diagnostic precision [9]. Nevertheless, implementation and impact evidence is still scarce in non-high-income countries, and diagnostic stewardship is another relevant concept that can decrease unnecessary testing and enable the suitable usage [8]. Since panels are defined in advance and can be adjusted to suit pathogens that are more prevalent in the North American/European environment, algorithms should be localized to regional epidemiology and incorporate a reflex/add-on testing [8,44]. There are also instances of missed clinically relevant organisms on predefined panels, which subsequently grow in culture and are absent in the multiplex targets. Hence, workflow tailored to LMICs should clearly incorporate the use of context-dependent ordering criteria with the help of local microbiologic maps if possible [44]. However, further research may be necessary due to its limitations in identifying specific fungi, such as *Cryptococcus neoformans or gatii* [6]. This is particularly noteworthy since Cryptococcus has been one of the analytes with a greater share of false negatives, which have also been noted in patients with positive antigen titers (including treated/cleared disease). This argues in favor of the relevance of CrAg-based pathways in comparison to multiplex PCR in the appropriate populations [6]. In line with the latter, initial assessments of some panels revealed diminished Cryptococcus target identification (e.g., 7/13 C. neoformans targets identified), which supports the requirement of parallel CrAg/culture verification where cryptococcosis is prevalent or suspected [24].

#### 4.2.3. Algorithm-Guided Testing—The Integration of Biochemical Markers

Algorithm-guided testing includes utilizing biomarkers found in CSF, especially CSF lactate. A CSF lactate threshold of more than 4.65 mmol/L was found to be a robust predictor of bacterial meningitis in one study, with a sensitivity of 81.5% and a specificity of 96.4% [44]. This implies that in cases of suspected community-acquired bacterial meningitis, high lactate levels could be a valid screening criterion or threshold to support and prioritize the use of expensive molecular diagnostics, especially when pre-test probability is high. However, future studies should validate the lactate-guided triage pathway in different subgroups, such as patients with immunosuppression.

### 4.3. Artificial Intelligence (AI) and Machine Learning (ML) in Meningitis Diagnostics

#### 4.3.1. Application of AI in Pathogen Detection—Metagenomics

New research has been emerging in the past few years about the use of artificial intelligence (AI) and machine learning (ML) in the diagnosis of meningitis. First, AI includes computer systems that would generally require human intelligence for task performance, such as data analysis, understanding speech, and predictions in management and risk stratification [45]. In this case, AI could be used for the interpretation of extensive diagnostic datasets, such as those of metagenomic next-generation sequencing (mNGS) [46].

ML, on the other hand, is considered a pillar of AI. As the name implies, it learns from large data patterns rather than being directly programmed for appropriate output. In this case, more learning occurs when more data are received [45]. This way, predictions are better, and so are management and risk stratifications. There are three types, and among them, deep learning is the most advanced for the interpretation of complex biological data while using artificial neural networks [45].

Metagenomic sequencing, as mentioned above, can be used in conjunction with AI/ML for the identification of a larger extent of pathogens, which may even be missed with cultures or PCR panels. In fact, such sequencing does not depend on organisms that were previously defined; rather, any type of RNA or microbial DNA from the CSF would be sequenced instead for analysis [47]. As the word “meta” in mNGS indicates, extensive genetic data is generated, so not every sequence can truly represent an active or real infection. There may be artifacts, background noise, or even contaminants.

Nevertheless, ML may be helpful to stratify the former from the latter [48]. In other words, real pathogens may be sorted away from contaminants, and pathogens may be ranked according to the likelihood of causing an active infection or disease.

Overall, mNGS heightens the detection of the disease, meaning that sensitivity is increased [49]. The latter was evidenced in a retrospective study, which compared mNGS to conventional methods, such as smears and cultures, where mNGS was found to be optimal for the detection of viral encephalitis and meningitis, along with a higher positive rate for patients who have bacterial meningitis. Overall, mNGS had a much better detection rate, which was relatively higher than for cultures [49]. Therefore, introducing it may also have a clinical impact in terms of management and risk stratification [47].

#### 4.3.2. Prognosis and Outcome Prediction

AI and ML are being used more frequently to predict prognosis, improve the interpretation of M/E panel data, and ultimately affect patient outcomes. In one study, patients with tuberculous meningitis had brain MRI scans, and a convolutional neural network (CNN) was used to predict bad outcomes, such as neurological outcomes or death [50]. Areas under the curve (AUC) were measured. The AUC in the non-imaging cohort (clinical with laboratory data) corresponded to an AUC of 71.2%, highlighting a decent prediction ability. As for the imaging-only cohort (brain MRI), it corresponded to a lesser AUC of 67.3%. However, their combination with CNNs had a superior AUC of 77.3% to the other two cohorts, indicating that MRI data interpretation with CNNs provides a good complementary predictive value, particularly in HIV-negative patients [50]. This method also made the model interpretable through saliency maps by emphasizing brain areas, such as the corpus callosum and Sylvian fissures, providing insights into disease pathophysiology [50].

Furthermore, researchers created an ensemble AI model that significantly outperformed clinicians in determining the cause of meningitis and encephalitis. The model outperformed both junior and senior neurologists with an outstanding accuracy of 89.09% and an F1 score of 0.8948 using data from within the first 24 h of a patient’s hospital stay [51]. This number alludes to a strong balance in precision and recall. In addition to providing a quick diagnosis, the system used explainable AI approaches to emphasize the important clinical indicators that guided its choice, such as mononuclear cell ratios and cerebrospinal fluid adenosine deaminase [51]. By guaranteeing that the appropriate treatment is administered at the most crucial moment, this skill allows physicians to start precisely targeted therapy earlier than ever before, significantly improving prognosis and patient outcomes.

## 5. Conclusions

To conclude, multiplex M/E panels can significantly save time in detecting pathogens. The latter enables more timely and specialized antimicrobial decisions, in part due to high levels of analytical sensitivity and specificity. These panels potentially have value in antimicrobial stewardship. To do so, they facilitate the earlier escalation or de-escalation of therapy. Nevertheless, even in confirmed cases of bacterial or fungal infections, evidence also points to limited effects on subsequent hospital outcomes, such as length of stay. In other words, their benefits are limited to clarifying the diagnosis and the optimization of antimicrobials, rather than pure clinical outcomes. In line with this, clinical judgment is always necessary, particularly when there is suspicion of rare, non-panel, and healthcare-associated pathogens. Possibly, algorithm-driven testing policies that use CSF biomarkers, including lactate-based strategies, could enhance the diagnostic accuracy [44]. Thus, further multicenter research is needed to clearly establish long-term clinical outcomes, cost-effectiveness, and utility in a wide range of patient populations, specifically in resource-limited environments. Development of the coverage of pathogens and the interpretation of results will be required to better define the clinical role of multiplex panels in the modern diagnosis of infections of the central nervous system.

## Figures and Tables

**Table 1:** Specificity and sensitivity of pathogens detection via the M/E panel in different studies and reviews.

Authors	Location	Sensitivity	Specificity	False Positives and Negatives
[9]	Eleven geographically distinct U.S. sites over a period of approximately eight months (February–September 2014).	100% for 9 over 14 pathogens; 95.7% for enteroviruses, 85.7% for HHV-6, 0% sensitivity for *Streptococcus agalactiae*.	More than 99.2% for most pathogens.	One false positive and false negative result for *Streptococcus agalactiae*, for a sensitivity.
[6]	Review article that included many centers, 56 centers in eight countries: Canada, Chile, India, Lithuania, Mexico, Peru, Russia, and the United States. Fifty-eight centers in seven countries: the United States, Canada, Argentina, Russia, India, Estonia, and Lithuania. A total of 77 centers in nine countries: Argentina, Brazil, Canada, Chile, Germany, Republic of South Africa, Spain, the United Kingdom, and the United States. A total of 28 centers in four countries.	Approximately 90%.	Approximately 97%.	False positives in low prevalence areas and false negatives for low viral or bacterial load.
[11]	Los Angeles County + University of Southern California Medical Center and Harbor-UCLA Medical Center are two tertiary cares	100% for bacteria, 50% for fungi such as *Cryptococcus neoformans.*	99.9% for bacteria, 100% for fungi.	Some positive cryptococal antigens and cultures had variable panel results.
[7]	Systematic review/study location not mentioned	More than 95%.	More than 99%.	Some false positives are present, false negatives for low viral or bacterial load.
[4]	Systematic review/study location not mentioned	Approximately 90%.	Approximately 97%.	Some false positives are present, false negatives for low viral or bacterial load.
[2]	Salt Lake City, USA	96.4%	95.24%	Some false positives and false negatives have been resolved via comparator PCR or sequencing.

HHV-6: Human Herpes Virus-6; PCR: Polymerase Chain Reaction.

**Table 2:** Comparison of false positives and negatives in different pathogens across studies.

Pathogen	Type of Error	High-Risk Pitfalls	Clinical Implications
HHV-6 [9]	False positive	Detection is usually an indication of infection in ciHHV-6 or of silent congenital infection, but not of acute disease.	Inappropriate antiviral use and diagnosis in neonates.
HSV-1/2 [9]	False positive	Four false-positive results in total.	Inappropriate antiviral use such as acyclovir.
Streptococcus pneumoniae [9,15]	False positive	Twelve false positives are high, mostly because of contamination.	Inappropriate antibacterial use, longer LOS.
Streptococcus agalactiae (GBS) [15]	False positive and negative	False positives (15.4% in one analysis) and false negatives, which are persistent and occasional, respectively.	Wrong or delayed treatment in neonates.
Cryptococcus neoformans [11]	False negative	Decreased sensitivity in low fungal burden or in treated patients. CrAg may stay positive despite negative panel results	Delayed diagnosis in immunocompromised patients.
Listeria monocytogenes [14]	False negative	Characteristic clinical presentation with limited panel sensitivity. A negative panel should not exclude high suspicion.	Delayed proper antimicrobial use.

ciHHV-6: Chromosomally Integrate Human Herpes Virus type 6; CrAg: Cryptococcal Antigen; GBS: Group-B Streptococcus; HHV-6: Human Herpes Virus type 6; HSV-1/2: Herpes Simplex Virus type 1 or 2; and LOS: Length of Stay.

**Table 3:** Comparison of the M/E panel and CSF culture for the detection of meningitis.

	CSF Culture	Multiplex PCR M/E Panel
Method [17,18]	Organisms are grown on media with MALDI, along with biochemical means.	Nucleic acid amplification with PCR detects a set of viral, fungal, and bacterial pathogens that are previously defined.
Turnaround time [18,19]	Approximately 24 to 72 h for bacteria, however it is longer for fungi and mycobacteria. Gram stain may allude to a preliminary result, which is faster.	Approximately 1 to 2 h in total (with the use of single-run cartridges).
Organisms detected [24]	May depend on culture techniques but can be any cultivable organism.	Limited to the pathogens that are previously defined, such as common ones. *Cryptococcus neoformans* may be present on some panels, however, unlisted, rare, or novel organisms are often absent.
Sensitivity for bacterial pathogens [15]	It is the gold standard; however, sensitivity depends on the use of prior antibiotics and bacterial load.	Sensitivity does not depend on the use of previous antibiotics. It is generally higher than cultures but may change depending on organisms.
Sensitivity for viral pathogens [4,18]	Culture is usually poor for viruses, and the latter is either slow or did not occurred usually.	Sensitivity is high compared to conventional methods including LDT PCR that are virus dependent. The panels can usually detect enteroviruses, HSV, VZV.
False positives [4,21]	Contamination may lead to false positives, but it is usually low for pathogens that are clinically significant.	If there are false positives, they generally require correlation with clinical data, which may impact treatment management and length of stay.
Pathogen load [9,22]	Cultures cannot provide Ct values, which correspond to the number of amplification cycles, which are required for the sample’s signal to cross the specific threshold that is set.	Some panels can include Cts, however reading is often qualitative (either a positive or a negative result)
Effect due to previous antibiotic use [15]	Antibiotics strongly affect culture results.	PCR usually detects nonviable RNA or DNA; thus it is more possible for it to stay positive, even after antibiotic use.
Effect on management [21]	It is essential for susceptibilities leading to target therapies.	Time for antibiotic use may be reduced, leading to shorter hospital stays and de-escalations in case of negative pathogens. However, stewardship varies, and impact may vary.
Cost [4]	Cost is usually lower; however, it requires developed and clean laboratory infrastructures. Per sample, the culture ends up being less expensive.	Each test costs higher (priced per cartridge or platform), depending on the equipment’s quality.
Implications and Regulations [4,23]	CSF cultures are widely available and are part of the standard care.	Some panels are FDA panels and used globally, however proper implementation needs stewardship with validation and algorithms for proper reporting and interpretation.

MALDI: Microscopic Identification or Matrix-assisted Laser Desorption/ionization; PCR: Polymerase Chain Reaction; LDT: Laboratory Developed Test; HSV: Herpes Simplex Virus; VZV: Varicella Zoster Virus; Ct: Cycle Threshold; RNA: Ribonucleic acid; DNA: Deoxyribonucleic acid; CSF: Cerebrospinal Fluid; and FDA: Food and Drug Administration.

## Data Availability

Data sharing does not apply to this article, as no new data were created or analyzed in this study.
